# Characterization of Port Bolivar Virus, a Novel Entomobirnavirus (*Birnaviridae*) Isolated from Mosquitoes Collected in East Texas, USA

**DOI:** 10.3390/v12040390

**Published:** 2020-03-31

**Authors:** Robert B. Tesh, Bethany G. Bolling, Hilda Guzman, Vsevolod L. Popov, Ashley Wilson, Steven G. Widen, Thomas G. Wood, Peter J. Walker, Nikos Vasilakis

**Affiliations:** 1 Department of Pathology, The University of Texas Medical Branch, Galveston, TX 77555-0609, USA; rtesh@utmb.edu (R.B.T.); rbtesh@comcast.net (H.G.); vpopov@utmb.edu (V.L.P.); 2 Center for Biodefense and Emerging Infectious Diseases, University of Texas Medical Branch, 301 University Blvd., Galveston, TX 77555-0609, USA; 3 Center for Tropical Diseases, University of Texas Medical Branch, 301 University Blvd., Galveston, TX 77555-0609, USA; 4 Institute for Human Infection and Immunity, University of Texas Medical Branch, 301 University Blvd., Galveston, TX 77555-0610, USA; 5 World Reference Center for Emerging Viruses and Arboviruses, University of Texas Medical Branch, 301 University Blvd., Galveston, TX 77555-0609, USA; Bethany.Bolling@dshs.texas.gov; 6 Galveston County Mosquito Control, 5115 Highway 3, Dickinson, TX 77539, USA; Ashley.Wilson@co.galveston.tx.us; 7 Department of Biochemistry and Molecular Biology, University of Texas Medical Branch, 301 University Blvd., Galveston, TX 77555, USA; sgwiden@utmb.edu (S.G.W.); TexRex47@comcast.net (T.G.W.); 8 School of Chemistry and Molecular Biosciences, The University of Queensland, St Lucia, QLD 4072, Australia; peter.walker@uq.edu.au

**Keywords:** *Birnaviridae*, *Entomobirnavirus*, Port Bolivar virus, mosquito viruses

## Abstract

This report describes and characterizes a novel entomobirnavirus, designated Port Bolivar virus (PTBV), that was isolated from a pool of *Aedes sollicitans* mosquitoes collected in a saltwater marsh in East Texas, USA. Full genome sequencing and phylogenetic analyses indicate that PTBV is distinct but genetically related to Drosophila X virus and mosquito X virus, which are assigned to species in the genus *Entomobirnavirus*, family *Birnaviridae*. PTBV produced cytopathic effect (CPE) in cultures of mosquito (C6/36) cells, but not in Vero cell cultures. Ultrastructural studies of PTBV in infected C6/36 cells demonstrated unenveloped virus particles about 55 nm in diameter.

## 1. Introduction

The family Birnaviridae consists of viruses with two segmented dsRNA genomes, that form icosahedral, non-enveloped single-shelled particles with a diameter of approximately 65 nm [1]. The family currently includes seven genera; members of three genera (*Aquabirnavirus, Avibunyavirus* and *Blosnavirus*) infect vertebrates, whereas members of four genera (*Entomobiravirus, Dronavirus, Ronavirus* and *Telnavirus*) infect invertebrates.

In the genus *Entomobirnavirus*, only two viruses have been classified taxonomically to date: Drosophila X virus (DXV; species *Drosophila X virus*) was detected in laboratory-reared fruit flies (*Drosophila melanogaster*) in France [2] and mosquito X virus (MoXV; species *Mosquito X virus*) was detected by metagenomic sequencing in mosquitoes (*Anopheles sinensis*) collected in China [3]. Four other viruses cluster phylogenetically with the entomobirnaviruses but have not yet been classified: Culex Y virus (CuYV) was isolated from mosquitoes (*Culex pipiens* complex) collected in Germany [4]; Eridge virus (ERV) was detected by metagenomic sequencing of fruit flies (*Drosophila immigrans*) collected in the United Kingdom [5]; culicine-associated Z virus (CaZV) was detected by metagenomic sequencing in mosquitoes (*Ochlerotatus caspius* and *Oc. detritus*) collected in France [6]; and Espirito Santo virus (ESV) was discovered in mosquito cell cultures (*Aedes albopictus*) during studies of dengue type 2 virus in Brazil [7]. In addition, Thirlmere virus, which was isolated in 1980 from a water sample collected in the UK [8], is related antigenically to DXV but no nucleotide sequence data have yet been reported. In this report, we describe and characterize a novel seventh entomobirnavirus, designated Port Bolivar virus (PTBV), which was isolated from *Aedes sollicitans* mosquitoes collected in 2013 near Port Bolivar, Texas, USA.

## 2. Materials and Methods

### 2.1. Study Area and Mosquito Collection

PTBV was isolated from mosquitoes collected in a salt marsh on the Bolivar Peninsula of Galveston County, Texas, USA. Galveston County is located in the southeastern part of Texas, along the Gulf of Mexico. The county has a total area of 2264 km^2^ of which 979 km^2^ is land and 1282 km^2^ (57%) is water. Fresh and saltwater lakes, marshes and rivers are scattered throughout the county, providing abundant breeding sites for mosquitoes. For this reason and because of the subtropical climate, mosquitoes are a significant pest problem, so the county has an active mosquito surveillance and control program. As part of its surveillance program, the Galveston County Mosquito Control Division (GCMCD) collects adult mosquitoes with CO_2_-baited CDC light traps placed at designated sites throughout the county to monitor species composition and abundance during most of the year. 

### 2.2. Culture Methods

Light traps were collected early each morning by GCMCD personnel and returned to their field laboratory where the mosquitoes were sorted on a chill table and separated into pools by species, sex and trap locality. Representative mosquito pools collected during July 2013 were frozen and transported on dry ice to the World Reference Center for Emerging Viruses and Arboviruses (WRCEVA), Department of Pathology, University of Texas Medical Branch (UTMB) in Galveston for virus isolation.

After thawing, each mosquito pool was homogenized using a TissueLyser (Qiagen, Hilde, Germany) in tubes with 1.5–2.0 mL of phosphate-buffered saline, pH 7.4, containing 10% fetal bovine serum, 1% penicillin–streptomycin–amphotericin stock (Sigma, St Louis, MO, USA) and several 3 mm stainless steel balls. After centrifugation, 150 μL of the supernatant was inoculated into separate 12.5 cm^2^ flask cultures of Vero E6 and *Aedes albopictus* C6/36 cells, originally obtained from the American Type Culture Collection, Manassas, VA. After adsorption for 2 h at 28 °C (C6/36) or 1 h at 37 °C (Vero), 5.0 mL of maintenance medium was added to each flask, and they were held in incubators at 28 and 37 °C, respectively. Cell cultures were examined regularly for evidence of viral cytopathic effect (CPE). The supernatant from a pool, designated GMC-202, produced CPE in the C6/36 cell culture beginning on day 6 post-inoculation; however, it did not produce visible CPE in the Vero cell culture.

### 2.3. Immunofluorescent Studies

A second flask of C6/36 cells was inoculated with culture fluid from the initial passage of GMC-202. Seven days after inoculation the infected cells were scraped from the surface of the culture flask and spotted onto Cell-Line 12-well glass slides (Thermo Fisher Scientific, Waltham, MA, USA) for examination by indirect fluorescent antibody tests (IFAT) [9], using hyperimmune mouse ascitic fluids prepared against eastern equine encephalitis (EEEV), Western equine encephalitis (WEEV), Saint Louis encephalitis (SLEV), West Nile (WNV), San Angelo (SAV) and Flanders (FLAV) viruses provided by WRCEVA.

### 2.4. Transmission Electron Microscopy (TEM)

For ultrastructural analysis, C6/36 cells infected with the second passage of GMC-202 were fixed for 1 h in a mixture of 2.5% formaldehyde prepared from paraformaldehyde powder and 0.1% glutaraldehyde in 0.05 M cacodylate buffer (pH 7.3), to which 0.01% picric acid and 0.03% CaCl_2_ were added. The monolayer was washed in 0.1 M cacodylate buffer, and cells were scraped off and processed further as a pellet. The pellet was post-fixed in 1% OsO_4_ in 0.1 M cacodylate buffer (pH 7.3) for 1 h, washed with distilled water and stained in block with 2% aqueous uranyl acetate for 20 min at 60 °C. The pellet was dehydrated in ethanol, processed through propylene oxide and embedded in Poly/Bed 812 (Polysciences, Warrington, PA, USA), as described previously [10]. Ultrathin sections were cut on a Leica EM UC7 microtome (Leica Microsystems, Buffalo Grove, II, USA), stained with lead citrate and examined in a Phillips 201 transmission electron microscope (FEI Phillips, Hillsboro, OR, USA) at 60 kV.

### 2.5. RNA Extraction and Next-Generation Sequencing

Fluid supernatants from cultures of infected C6/36 cells were used for RNA extraction and sequencing. Supernatants were harvested on day 6 post-infection and clarified by low-speed centrifugation (2000× *g*, 10 min at 4 °C). One milliliter of clarified supernatant was treated with a cocktail of DNases (14 U Turbo DNase (Ambion, Austin, TX, USA), 20 U Benzonase (EMD Millipore, Billerica, MA, USA) and 20 U RNase One (Promega, Madison, WI, USA) for 1 h at 37°C. Viral RNA was then extracted using Trizol and resuspended in 50 μL RNase/DNase and protease-free water (Ambion, Austin, TX, USA). Viral RNA (~0.9 µg) was fragmented by incubation at 94°C for 8 min in 19.5 µL of fragmentation buffer (Illumina 15016648). Sequencing libraries were prepared from the sample RNAs using an Illumina TruSeq RNA v2 kit following the manufacturer’s protocol. The samples were sequenced on a HiSeq 1000 using the High-Output 2 × 50 paired-end protocol. Reads in fastq format were quality-filtered, and any adapter sequences were removed, using Trimmomatic (v0.17) [11] software. The de novo assembly program ABySS (v1.3.7) [12] was used to assemble the reads into contigs, using several different sets of reads, and kmer values from 20 to 40. Blastn and blastx searches with contigs over 400 bases were used to identify the viral segments. All other contigs were from host RNA. Contigs covering nearly the full length of the viral segments were obtained from 1 million read pairs and kmer values of 25 and 38 for the two segments. Reads were mapped back to the viral contigs using bowtie2 (v2.1.0) [13] and visualized with the Integrated Genomics Viewer (v2.3.26) [14] to verify that the assembled contigs were correct. There were 7.2 million paired reads after filtering and ~1.5% (109,537) mapped to the viral contigs. Raw sequencing data are available upon request to the corresponding author.

### 2.6. Phylogenetic Analysis

An alignment of complete birnavirus VP1 protein (RdRp) amino acid sequences was created using ClustalW in MEGA version 7.0, and phylogenetically informative sites were selected using Gblocks. The resulting alignment comprising 274 amino acids was used to infer phylogenetic relationships in MEGA 7.0 using the Maximum Likelihood method based on the Whelan and Goldman + Frequency model of amino acid substitution [15]. Initial tree(s) for the heuristic search were obtained automatically by applying Neighbor-Join and BioNJ algorithms to a matrix of pairwise distances estimated using a JTT model [16], and then selecting the topology with superior log likelihood value. The phylogenetic robustness of each node was determined using 1000 bootstrap replicates. Trees were annotated using Figtree version 1.4.2 (http://tree.bio.ed.ac.uk/software/figtree). 

## 3. Results

### 3.1. Virus Isolation

**Figure 1:**
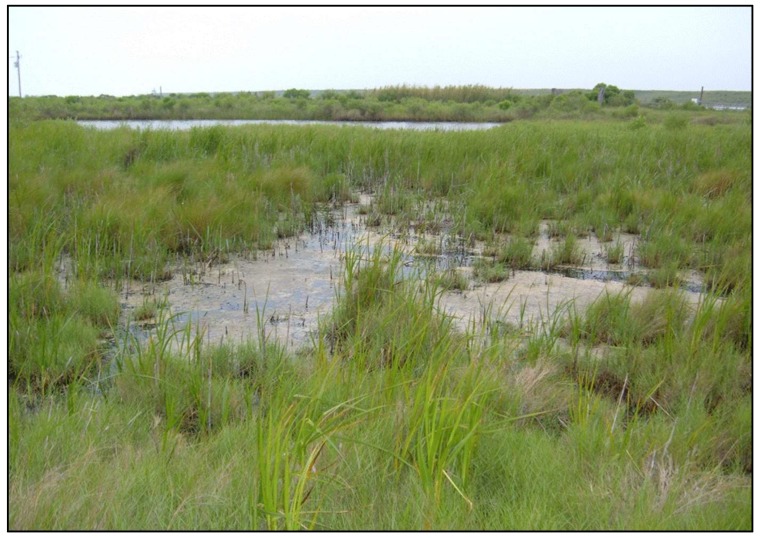
A saltwater marsh near Port Bolivar, Texas, where the infected *Aedes sollicitans* mosquitoes were collected. (Courtesy of Galveston County Mosquito Control).

Mosquito pool GMC-202 consisted of 40 non-blooded, female *Aedes sollicitans* collected in a light trap placed in a saltwater marsh (Figure 1) near the town of Port Bolivar, Galveston County, Texas (Segment A) on the evening of 25 July 2013. The mosquito pool homogenate produced moderate cytopathic effect (CPE) in the C6/36 cell culture beginning 6 days post-inoculation. No CPE was observed in Vero cells inoculated with GMC-202 after 14 days.

### 3.2. Immunofluorescent Studies

IFATs performed on the GMC-202-infected C6/36 cells were negative with mouse hyperimmune ascitic fluids (MIAFs), prepared against EEEV, WEEV, SLEV, WNV, SAV and FLAV and used at a 1:20 dilution (data not shown). These six agents are the most common mosquito-borne arboviruses recovered in East Texas. 

### 3.3. Transmission Electron Microscopy (TEM)

**Figure 2:**
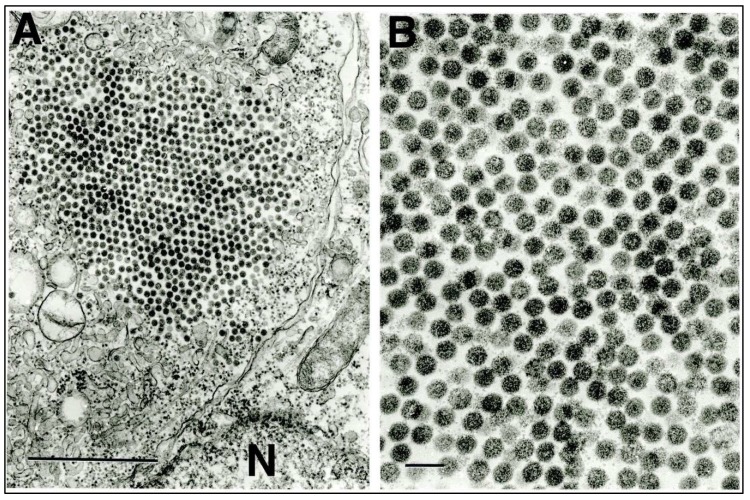
Ultrastructure of entomobirnavirus GMC-202 in C6/36 mosquito cells. (**A**) Paracrystalline agglomeration of virus particles in the cell cytosol. N = nucleus of adjacent cell. Bar = 1 μ. (**B**) Higher-power image of the agglomeration showing the details of virion ultrastructure. Bar = 100 nm.

In ultrathin sections of C6/36 cells infected with GMC-202, individual unenveloped virus particles about 55 nm in diameter were observed either scattered free in the cytosol or as paracrystalline agglomerates (Figure 2A,B).

### 3.4. Genomic Characterization

**Figure 3:**
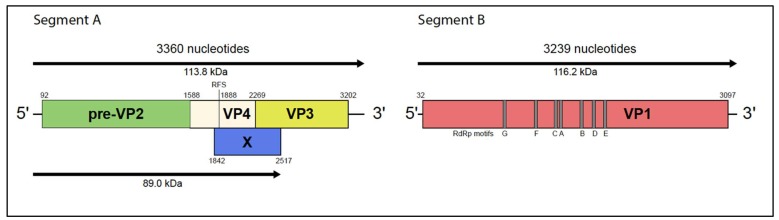
Genome organization of Port Bolivar virus.

The PTBV genome comprises two segments of double-stranded RNA (Figure 3). As in other entomobirnaviruses, segment A (3360 nt) contains a long open reading frame (ORF) encoding a 113.8 kDa polyprotein (pre-VP2-VP4-VP3) that is co-translationally processed by autocatalysis to generate three polypeptides: pre-VP2 (54.6 kDa), which is further processed to generate major capsid protein VP2; core protein VP3 (35.1 kDa); and serine/lysine protease VP4 (24.2 kDa) [17]. An alternative long ORF (X) in segment A commences 46 nt downstream of a putative –1 ribosomal frame-shift site (UUUUUUAA) that is predicted to generate an 89.0 kDa protein (pre-VP2-VP4N-X) [2]. PTBV RNA segment B (3239 nt) contains a single long ORF encoding VP1, the 116.2 kDa RNA-dependent RNA polymerase (RdRp). PTBV VP1 includes all seven recognized sequence motifs (A–G) in the ‘palm’ and ‘fingers’ domains that form the distinctive topology of the birnavirus RdRp [18]. The PTBV RdRp also shares with other birnaviruses the unusual active site motif (AND) rather than the G/SDD motif that is characteristic of most (+) ssRNA viruses [18,19]. 

The PTBV genome architecture is similar to that of DXV and other entomobirnaviruses [1]. In particular, segment A encodes a polyprotein (pre-VP2-VP4-VP3) in one long ORF and an alternative ORF (X) that appears likely to be expressed by –1 ribosomal frameshift at a “slippery” site (UUUUUUAA), which is conserved amongst entomobirnaviruses and has been recognized as a particularly shift-prone sequence [2,4]. Although the DXV polyprotein (pre-VP2-VP4-VP3) has been shown to be processed autocatalytically by the VP4 serine-lysine protease [17], the pre-VP2-VP4N-X protein generated by –1 frameshift would lack two critical elements of the VP4 protease active site [20]. As such, it is unlikely to be processed autocatalytically. However, as shown for several birnaviruses, maturation of VP2 involves further processing of the C-terminal region of pre-VP2, most likely by cellular proteases [21,22,23]. Therefore, subsequent processing of PTBV pre-VP2-VP4N-X to generate VP2 and VP4N-X cannot be excluded.

### 3.5. Amino Acid Sequence Identities

Pairwise amino acid sequence identities (p-distances) were estimated in MEGA 7 from a ClustalW alignment of available birnavirus VP1 (RdRp) proteins (Table 1) and birnavirus polyproteins (Table 2). In VP1, PTBV showed highest sequence identity with entomobirnaviruses and is most closely related to CaZV (87.8% identity) and most distantly related to ERV (69.4% identity). Amongst entomobirnavirus pre-VP2-VP4-VP3 polyproteins, PTBV is most closely related to CaZV (91.7% identity) and most distantly related to DXV (69.6% identity).

### 3.6. Phylogenetic Analysis

**Figure 4:**
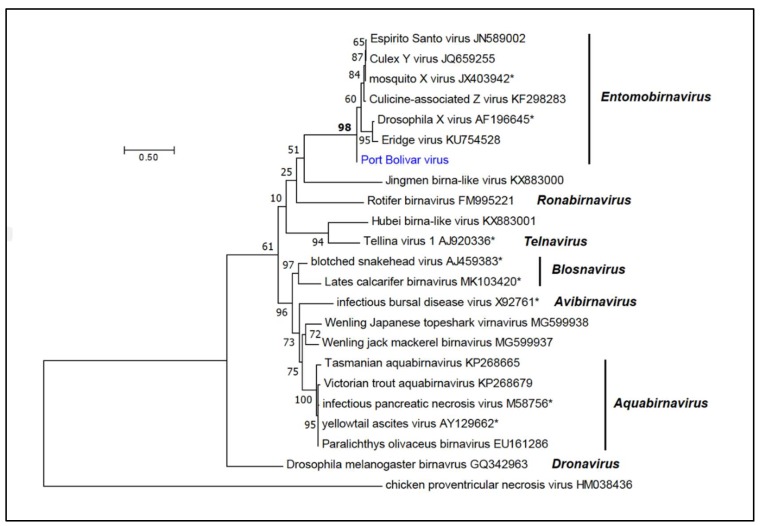
Phylogenetic relationships of Port Bolivar virus. An unrooted phylogenetic tree was inferred by using the Maximum Likelihood method from a ClustalW alignment of complete VP1 protein (RdRp) amino acid sequences of Port Bolivar virus (PTBV) and 22 birnaviruses. Phylogenetically informative sites were selected from the alignment using Gblocks, resulting in 274 positions in the final dataset. The tree with the highest log likelihood (−6338.33) is shown. The tree was drawn to scale, with branch lengths measured in the number of substitutions per site. Bootstrap values (100 iterations) are shown for each node. All seven established genera are shown in bold italics; those viruses which have currently been assigned to species are also shown.

Phylogenetic analysis conducted using a ClustalW alignment of VP1 (RdRp) amino acid sequences of PTBV and 22 birnaviruses indicated that it clustered with strong bootstrap support (BSP = 98%) with the entomobirnaviruses (Figure 4).

## 4. Discussion

Along with CuYY, PTBV is the second entomobirnavirus to be isolated from free-living mosquitoes (Table 3) [4]. Two others, CaZV and MXV, were detected by next-generation sequencing (without isolation) of field-collected mosquitoes [3,6]. The remaining four known entomobirnaviruses (DXV, ERV, ESV and Thirlmere virus) were isolated from or were detected by next-generation sequencing of insect cell lines, or in a water sample passed in insect cell cultures [2,5,7]. For this reason, there is some uncertainty as to their origin. For example, ESV was discovered during purification of a strain of dengue virus type 2 (DENV-2) that was originally isolated from a Brazilian dengue patient and had been passaged three times in C6/36 cell cultures [7]. DXV, the prototype of the genus *Entomobirnavirus*, was discovered under similar circumstances. DXV was isolated as a contaminant during infection studies with sigma virus (genus *Sigmavirus: Rhabdoviridae*) [2]. 

Currently, only two species (*Drosophila X virus* and *Mosquito X virus*) have been assigned in the genus *Entomobirnavirus* by the International Committee on Taxonomy of Viruses (ICTV), and no formal species demarcation criteria appear to have been published. Phylogenetically, PTBV clearly falls within this genus and, based upon amino acid sequence divergence in VP1 RdRp and the VP2-VP4-VP3 polyprotein, it is clearly distinct from both DXV (27.7% and 30.4% divergence, respectively) and MXV (14.1% and 13.8% divergence, respectively) (Figure 4). Although PTBV is most closely related to CaZV, these viruses have been detected in culicine mosquitoes of different species from geographically distant locations (*Aedes sollicitans* in Texas and *Ochlerotatus* spp. in France, respectively) and are sufficiently divergent in amino acid sequence (12.2% divergence in VP1; 8.3% divergence in VP2-VP4-VP3) to be assigned to distinct new entomobirnavirus species.

To date, the strongest association of entomobirnaviruses has been with mosquitoes; but the effects of this group of viruses on their mosquito hosts are still unknown. With the current interest in mosquito microbiomes, this should be an area for future study. Also, given their wide geographic distribution and the diversity of mosquito hosts, it seems likely that many other entomobirnaviruses exist in nature in mosquitoes and possibly other insects and arthropods.

## Figures and Tables

**Table 1:** Percentage amino acid sequence identities (p-distance) of a ClustalW alignment of birnavirus VP1 (RdRp) proteins.

Genus	Virus	PTBV	CaZV	CuYV	MXV	ESV	DXV	ERV	IPNV	TABV	VTABV	IBDV	BSHV	LCBV	RBV	DBV	TV-1	JmBLV
*Entomobirnavirus*	PTBV																	
	CaZV	87.8																
	CuYV	85.8	92.2															
	MXV *	85.9	92.5	98.5														
	ESV	86.1	92.1	98.8	98.1													
	DXV *	72.3	72.5	72.5	72.8	72.5												
	ERV	69.4	71.3	71.6	71.7	71.6	84.7											
*Aquabirnavirus*	IPNV *	28.4	28.4	28.3	28.1	28.1	29.2	28.7										
	TABV	28.6	28.0	28.4	28.3	28.3	28.1	28.0	90.8									
	VTABV	29.0	29.0	28.7	28.6	28.6	29.4	29.1	94.5	90.4								
*Avibirnavirus*	IBDV *	29.1	28.8	28.1	28.1	27.9	28.1	27.7	48.0	47.4	47.4							
*Blosnavirus*	BSHV *	29.6	29.8	29.9	30.1	29.8	29.5	29.8	49.2	48.2	49.0	52.3						
	LCBV *	28.6	29.0	29.5	29.6	29.1	28.6	28.4	50.4	48.9	49.7	51.0	62.2					
*Ronavirus*	RBV *	26.1	25.1	24.7	25.0	24.9	25.0	24.9	30.9	31.3	31.0	30.2	31.7	30.7				
*Dronavirus*	DBV *	24.5	23.4	23.9	24.2	23.9	23.4	22.4	29.5	29.2	29.8	27.7	29.6	29.6	24.6			
*Telnavirus*	TV-1 *	24.5	24.2	23.9	23.5	23.6	25.0	25.1	30.3	30.5	30.1	31.1	31.8	33.3	28.7	25.0		
unassigned	JmBLV	28.1	27.3	27.3	27.3	27.3	27.3	28.0	31.7	31.0	31.1	30.2	30.6	30.7	26.9	23.2	26.4	

* Only these viruses are currently formally classified to species.

**Table 2:** Percentage amino acid sequence identities (p-distance) of a ClustalW alignment of birnavirus VP2-VP4-VP3 polyproteins.

Genus	Virus	PTBV	CaZV	CuYV	MXV	ESV	DXV	ERV	IPNV	TABV	VTABV	IBDV	BSHV	LCBV	RBV	DBV	TV-1
*Entomobirnavirus*	PTBV																
	CaZV	91.7															
	CuYV	85.9	89.2														
	MXV *	86.2	89.7	98.4													
	ESV	85.1	88.1	98.4	97.3												
	DXV *	69.6	70.5	69.3	68.8	68.9											
	ERV	71.3	72.7	70.7	70.4	70.4	86.1										
*Aquabirnavirus*	IPNV *	25.2	25.2	25.0	24.9	24.8	24.8	25.6									
	TABV	25.1	24.5	24.4	24.4	24.2	24.3	25.1	84.7								
	VTABV	25.3	25.0	24.5	24.4	24.4	24.3	25.3	88.8	86.8							
*Avibirnavirus*	IBDV *	25.1	24.8	25.2	25.2	25.0	25.6	27.0	35.5	35.5	36.1	###					
*Blosnavirus*	BSHV *	26.6	26.6	26.4	26.3	26.4	25.6	27.7	35.3	36.2	35.8	39.8					
	LCBV *	24.5	25.0	25.0	24.9	24.9	23.8	25.0	34.5	34.8	33.8	39.6	51.5				
*Ronavirus*	RBV *	24.9	24.7	25.0	24.5	24.7	25.7	26.5	27.5	26.6	27.0	27.7	28.2	26.3			
*Dronavirus*	DBV *	24.9	24.5	24.0	24.0	24.0	24.3	24.0	27.5	27.7	28.7	29.2	27.4	27.7	25.7		
*Telnavirus*	TV-1 *	21.5	21.6	20.9	21.1	20.5	22.6	22.6	23.8	24.3	22.9	26.0	26.7	26.2	25.7	23.1	

* Only these viruses are currently formally classified to species.

**Table 3:** Names, original source, locality, and accession numbers of currently recognized entomobirnaviruses *.

Virus Name (Abbreviation)	Source	Locality	Accession Numbers	Reference
Drosophila X virus (DXV)	*Drosophila melanogaster* cell culture	France	U60650; AF196645	[2]
Eridge virus (ERV)	*D. melanogaster* cell culture	UK	KU754527; KU754528	[5]
Culicine-associated Z virus (CaZV)	*Ochlerotatus caspius* and *Oc. detritus*	France	KF298271; KF298272	[6]
Espirito Santo virus (ESV)	*Aedes albopictus* cell culture	Brazil	JN589003; NJ589002	[7]
Mosquito X virus (MXV)	*Anopheles sinensis*	China	JX403941; JX403942	[3]
Culex Y virus (CuYV)	*Culex pipiens* complex	Germany	JQ659254; JQ659255	[4]
Thirlmere virus	Water	UK	Not available	[8]
Port Bolivar virus (PTBV)	*Aedes sollcitans*	USA	MT263973 MT263974	Present paper

* Entomobirnaviruses reported as of February 2020.
